# Comparative Social Life Cycle Assessment of Two Biomass-to-Electricity Systems

**DOI:** 10.3390/ijerph18094918

**Published:** 2021-05-05

**Authors:** Mario Martín-Gamboa, Paula Quinteiro, Ana Cláudia Dias, Diego Iribarren

**Affiliations:** 1 Chemical and Environmental Engineering Group, Rey Juan Carlos University, 28933 Móstoles, Spain; 2 Centre for Environmental and Marine Studies (CESAM), Department of Environment and Planning, University of Aveiro, Campus Universitário de Santiago, 3810-193 Aveiro, Portugal; p.sofia@ua.pt (P.Q.); acdias@ua.pt (A.C.D.); 3 Systems Analysis Unit, IMDEA Energy, 28935 Móstoles, Spain; diego.iribarren@imdea.org

**Keywords:** bioenergy, electricity, life cycle assessment, social risk, sustainability

## Abstract

Biomass plays a fundamental role in numerous decarbonisation strategies that seek to mitigate the short- and long-term effects of climate change. Within this context, decision-makers’ choices need to comprehensively consider potential sustainability effects associated with bioenergy systems. In particular, due to the lack of studies addressing the social sustainability of bioelectricity, the present work applies the Social Life Cycle Assessment (S-LCA) methodology to compare the social performance of two biomass-to-electricity systems located in Portugal based on either fluidised-bed or grate furnace technology. S-LCA involves a comprehensive approach for holistic evaluation and data interpretation of social aspects. Six social indicators were benchmarked: child labour, forced labour, gender wage gap, women in the sectoral labour force, health expenditure, and contribution to economic development. The results show that the implementation of fluidised-bed furnaces as a more efficient conversion technology could reduce by 15–19% the selected negative social impacts, except women in the sectoral labour force. When enlarging the interpretation to a sustainability perspective, the general suitability of the fluidised-bed furnace system would be further emphasised under environmental aspects while jointly providing valuable insights for informed decision-making and sustainability reporting.

## 1. Introduction

A considerable number of countries have boosted the implementation of national targets to become carbon neutral by 2050 [1]. The pivotal point of the related national plans is the transition towards low-carbon renewable energy technologies [2]. According to the International Energy Agency (IEA) [3], global energy-related carbon dioxide (CO_2_) emissions reached around 33 Gt in 2019. Despite a fall in the energy-related CO_2_ emissions in 2020 (attributable to the economic and behavioural effects of the coronavirus pandemic), this trend could be reversed in 2021 with a recovery in economic activity [4]. In order for this trend to become an actual turning point, national plans towards carbon neutrality should focus on reducing energy demands and increasing energy efficiency while the progressive reduction in the costs of low-carbon energy technologies makes their deployment attractive.

Renewable energy systems based on biomass processing play a fundamental role in national energy roadmaps [2,5]. In fact, systems for bioenergy/biofuel production have been widely deployed in the European Union during the last years to meet the targets of the Renewable Energy Directive [1]. Furthermore, the deployment of bioenergy systems is closely linked to several Sustainable Development Goals (SDGs) such as SDG7 (affordable and clean energy) and SDG13 (climate action) [6]. In particular, bioenergy systems are interesting for power purposes due to their flexibility and ability to provide energy that can be dispatched to balance dynamic demands [7]. From the beginning of the 21st century, technological advancements in terms of efficiency, new system concepts and/or alternative biomass feedstocks have boosted the use of bioenergy systems for power generation, partly decarbonising economies and societies. As bioenergy is included in a considerable number of national energy strategies, decision-makers need to be aware of the environmental, economic, and socials aspects associated with the technological advancements and subsequent implementation of bioenergy technologies. To that end, thorough sustainability analyses of bioenergy systems should be conducted using holistic methodologies, especially life-cycle approaches [8].

In the last years, a considerable number of life-cycle studies on biomass-to-electricity systems have been published, usually focusing on the economic and/or environmental aspects of one or more technologies. For instance, regarding conventional life cycle costing (LCC) studies, Cardoso et al. [9] carried out a techno-economic analysis of an 11 MW gasification power plant using forest biomass blends in Portugal. The results—in terms of net present value (NPV), internal rate of return (IRR), and payback period (PBP)—show the viability of the project, although the economic performance strongly depends on revenues from electricity sales regulated by uncertain tariffs and reimbursements. Jongdeepaisal and Nasu [10] developed a method based on hybrid input–output (I-O) analysis to evaluate the economic impact of a biomass power plant, assessing the influence of the biomass power plant on economic sectors and local economy related to resource extraction. Jin et al. [11] summarised technical and economic aspects of different gasification technologies as an alternative technique for electricity production, providing a better understanding of the advantages and disadvantages of biomass gasifiers to produce low-tar and high-caloric-value syngas for power generation, especially in rural areas. Kaoma and Gheewala [12] conducted a techno-economic assessment of bioenergy options for the provision of electricity services in rural areas of Zambia, finding that none of the biomass-based electricity production technologies considered in the study would be cost-competitive with the (subsidised) national electricity tariffs.

Regarding environmental life cycle assessment (LCA), Muench and Guenther [8] provide an overview of the environmental impacts of biomass-based electricity and heat, showing the high influence of methodological choices on the results. Additionally, Muench [13] carried out an exploratory analysis of life-cycle studies to identify the greenhouse gas mitigation potential of different biomass-to-electricity systems, finding that this type of system can be an appropriate choice and LCA is a suitable tool for sustainability assessment and informed decision-making. Similarly, Kadiyala et al. [14] evaluated the life-cycle greenhouse gas emissions associated with bioelectricity systems using different biomass feedstock categories. Paletto et al. [15] also conducted a comprehensive review of environmental LCA studies of biomass power plants, identifying the size of the plant and the feedstock as key variables that influence many environmental impacts. 

Several studies present a joint evaluation of environmental and economic aspects of power generation systems based on biomass [16,17]. In contrast, no attention is typically paid to the social dimension of biomass-to-electricity systems, barely finding a couple of studies that assess the social performance of biomass power systems from a life-cycle perspective [18,19]. Hence, it is essential to fill the scientific gap in social life-cycle studies of biomass-to electricity systems in order to avoid burden shifting along sustainability dimensions, ensuring that the technological improvements developed so far and their implementation also lead to a viable social performance and thus strengthening the alignment with SDGs such as SDG1 (no poverty), SDG3 (good health and well-being), SDG5 (gender equality), SDG8 (decent work and economic growth), and SDG10 (reduced inequalities). In other words, the consideration of social-oriented SDGs would be enhanced besides that of techno-economic and environmental ones relevant to bioenergy systems [6]. To shed light on this relevant topic, the present study applies the Social Life Cycle Assessment (S-LCA) methodology [20,21] to compare the performance of two biomass-to-electricity systems located in Portugal based on the use of either fluidised-bed or grate furnace technology for energy conversion. This methodology aims to thoroughly evaluate the potential social impacts and benefits of product systems, thus providing a sound basis for decision-makers to move towards social responsibility and well-being. The S-LCA guidelines launched by the UNEP’s Life Cycle Initiative in 2009 [20] originally set the pillars of the methodology, and they were updated in 2020 to provide a more practical framework [21]. The article is organised as follows: Section 2 describes the S-LCA methodological framework, the bioelectricity systems, the social life cycle inventory (S-LCI) data, and the social life cycle impact assessment (S-LCIA) method; Section 3 provides the S-LCIA scores of both systems, and a joint interpretation and benchmarking of their sustainability performance; and Section 4 presents the conclusions of the study as well as future research directions.

## 2. Materials and Methods 

The main objective of this work is to answer the following research question: could technological improvements in biomass-to-electricity systems potentially lead to a reduction in social risks across the supply chain? This was addressed through S-LCA, completing the sustainability picture of a bioelectricity system based on fluidised-bed furnace technology [22] and comparing its performance with that of a bioelectricity system using grate furnace technology [19]. The updated S-LCA guidelines by the UNEP’s Life Cycle Initiative [21] were used as a key reference at the methodological level.

### 2.1. S-LCA Framework

**Figure 1:**
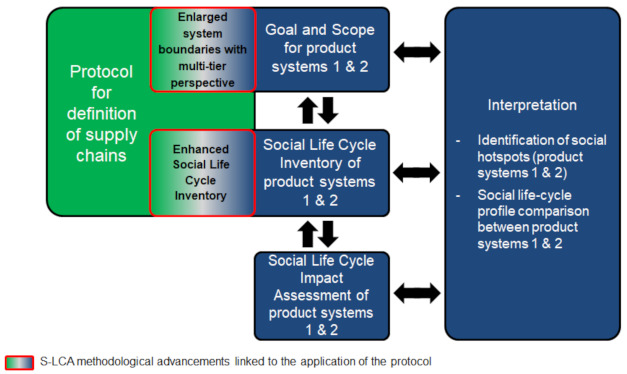
S-LCA framework for the comparative assessment of two biomass-to-electricity systems.

S-LCA is—to a certain extent—based on the standardised framework set in ISO 14040 and 14044 for environmental LCA, including four interrelated phases [23,24]. Figure 1 shows the four phases of the S-LCA methodology, adapted to the focus of the study: the comparative S-LCA of two biomass-to-electricity systems. Given the importance of country- and sector-specific data in S-LCA [21], the S-LCA methodology was combined with a general protocol for the definition of supply chains which allows analysts to identify representative countries of origin for the unit processes involved in a product system [19]. Such a complete picture of a product’s supply chain facilitates the identification of social risks, which would remain opaque without a multi-tier structure (e.g., social risks associated with unit processes located in developing countries).

The first step of the S-LCA methodology corresponds to goal and scope definition. In this step, the purpose of the study and the functional unit (FU) used to quantify the function of each product system should be clearly set. Furthermore, all the unit processes included in the analysis should be identified, defining the boundaries of each system. The definition of the boundaries still presents certain methodological fragility due to the low maturity of the S-LCA methodology [25,26], which was mitigated by using the above-mentioned protocol for supply chain definition through a procedure that jointly uses life cycle inventory (LCI) and trade databases [19].

S-LCI analysis is the second step of the S-LCA methodology, involving data collection for the unit processes within the boundaries of each product system. In this regard, it should be noted that the use of the supply chain definition protocol secondly enhances this step because of the need for data collection for an exhaustive list of unit processes. Working hours per FU were used in this work to ultimately measure activity variables [19,27,28]. Specific details on data acquisition can be found in Section 2.3. 

S-LCIA constitutes the third step of the methodology, dealing with the evaluation of the potential social impacts associated with the supply chain of each product system. To that end, activity variables are transformed into potential social impacts by using an impact assessment method. There are two main families of impact assessment approaches: the Reference Scale Approach (which uses performance reference points to assess the social performance of each product system) and the Impact Pathway Approach (which uses characterisation models to represent impact pathways and evaluate potential social impacts) [26,29,30]. In the present study, the first type of impact assessment approach was adopted by using an S-LCA database analysis (see Section 2.4).

Interpretation is the final step of the S-LCA methodology, in which the results of the previous steps are reviewed and discussed in depth to provide conclusions and recommendations for informed decision-making. As observed in Figure 1, this work addresses both the identification of social hotspots and the comparison of the social life-cycle profile of each product system.

### 2.2. Definition of the Case Study

**Figure 2:**
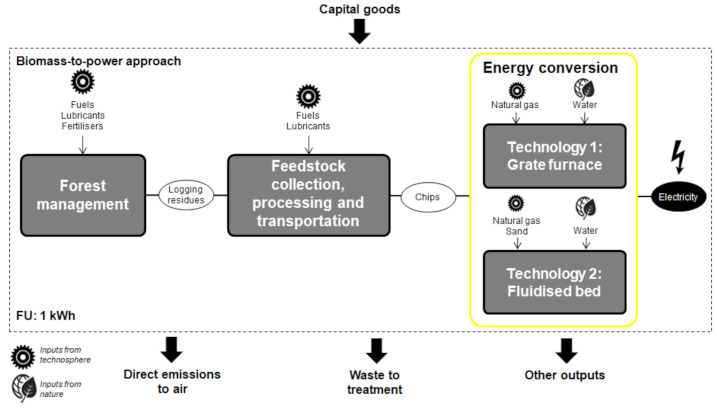
Boundaries of the biomass-to-electricity systems.

The goal of the S-LCA study is the identification of social hotspots across the supply chain of a bioelectricity system based on fluidised-bed furnace technology, and the comparison of its social life-cycle performance with that of a bioelectricity system based on grate furnace technology [19]. In both cases, the fuel is chips from eucalyptus logging residues produced in Portugal. The FU of both systems was defined as 1 kWh of electricity delivered to the grid. The boundaries (Figure 2) include three blocks: (i) forest management, (ii) feedstock collection, processing, and transportation, and (iii) energy conversion. The forest management stage includes infrastructure establishment, site preparation, stand establishment, stand tending, and tree felling. A high-intensity management for eucalyptus stands, relying on best management practices, was considered [31]. Eucalyptus stands are managed as coppiced stands in three successive rotations, each one of 12 years [31,32].

At the feedstock collection, processing, and transportation stage, the logging residues are collected with forwarders and transported to a terminal for chipping [33], being then transported to the power plant. The energy conversion stage is the distinguishing block in the two bioelectricity systems, involving chip combustion in either a grate furnace power plant or a fluidised-bed furnace power plant. Table 1 gathers key technical and socio-economic features related to the energy conversion stage of each bioelectricity system [22,34,35]. As observed in Figure 2, capital goods (i.e., buildings, machinery, and equipment) and their respective supply chains were included in the boundaries of both biomass-to-electricity systems. Further details on both biomass-to-electricity systems can be found in da Costa et al. [22] and Martín-Gamboa et al. [19].

### 2.3. Social Life Cycle Inventory

This section focuses on S-LCI, while conventional inventory data for environmental assessment—beyond data in Table 1—are readily available in da Costa et al. [22]. S-LCI data of the bioelectricity system involving grate furnace technology were directly retrieved from Martín-Gamboa et al. [19]. The same data acquisition procedures and sources were adopted for the bioelectricity system based on fluidised-bed furnace technology. Hence, initial foreground information was taken from da Costa et al. [22] while initial information associated with background processes was retrieved from the ecoinvent database v3.7.1 using the cut-off system model [36]. The UN Comtrade database [37] was used as a source of information to define the location of the unit processes at the country level. The translation of foreground inventory data into economic flows to build the S-LCI was based on levelised cost analysis [19,27,28], while global databases were used to estimate the economic value of background commodity flows [38]. Concerning the quantification of working hours, those associated with the operation of the bioelectricity plant were based on ILOSTAT [39], while those related to biomass production and processing were based on site-specific data available in the scientific literature [31,32,40]. Additionally, PSILCA—an S-LCA dedicated database—was used to estimate the working hours associated with background processes by considering specific countries and sectors as well as the related economic flows [41]. The use of such a database allows a thorough social study while providing transparent documentation on data sources and social risk levels. In this sense, the data-collection approaches presented in this section for S-LCI construction are in agreement with the current S-LCA guidelines [21].

**Figure 3:**
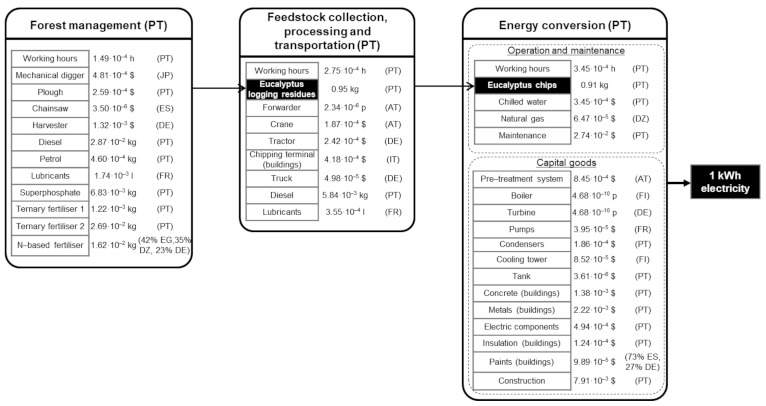
S-LCI data of the main blocks in the bioelectricity system based on fluidised-bed technology.

**Figure 4:**
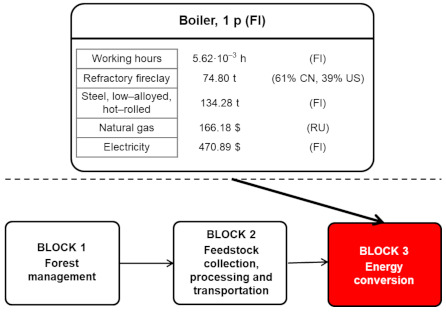
S-LCI data of the fluidised-bed boiler included in the energy conversion block.

**Figure 5:**
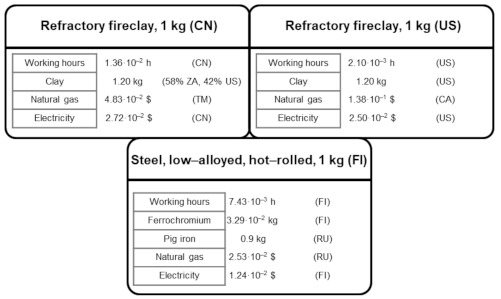
S-LCI data of refractory fireclay and steel for fluidised-bed boiler manufacturing.

**Figure 6:**
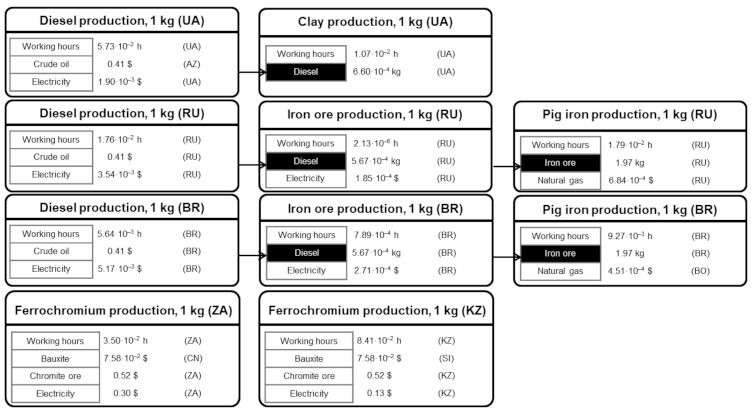
S-LCI data of unit processes associated with fireclay and steel for boiler manufacturing.

Figure 3 shows the main S-LCI data of the biomass-to-electricity system based on fluidised-bed furnace technology, while Figure 4, Figure 5 and Figure 6 further specify the S-LCI data of the fluidised-bed boiler (the only equipment that is different from the system based on grate furnace technology) and the background processes associated with its manufacturing in Finland. Each small box in Figure 3, Figure 4, Figure 5 and Figure 6 can be understood as a separate plant or business entity within the supply chain of the bioelectricity system [27,28]. In addition to inventory data (working hours as well as economic and non-economic flows), these figures include the protocol-based identification of the countries involved within the supply chain. As in the case of the biomass-to-electricity system based on grate furnace technology [19], the product system encompasses more than 400 processes within seven tiers of the supply chain, which stresses the thoroughness attained by using the supply chain definition protocol.

### 2.4. Social Life Cycle Impact Assessment Framework

PSILCA was also used as the impact assessment method [42]. It is one of the most common S-LCIA frameworks applied in the literature [25]. PSILCA provides statistical data for 88 indicators under 25 subcategories. Regarding these indicators, data are provided as risks associated with country-specific sectors, with a scale that ranges from no risk to very high risk. For the sake of clarity, social risks are generally understood as the probability of occurrence of adverse social effects on stakeholders due to the activities involved in the supply chain of a product system. In line with previous studies on energy systems [19,27,28,43], the following social indicators were evaluated: total child labour, frequency of forced labour, gender wage gap, women in the sectoral labour force, health expenditure, and contribution to economic development. This set of indicators involves risks that affect basic social pillars such as the defence of human rights, as well as a positive social impact (contribution to economic development) [42]. 

## 3. Results and Discussion

This section presents the S-LCIA results of the biomass-to-electricity system based on fluidised-bed furnace technology (Section 3.1), as well as their comparison with those of the bioelectricity system based on grate furnace technology (Section 3.2). Furthermore, Section 3.3 enlarges the comparison of both systems from a sustainability perspective.

### 3.1. Social Life Cycle Impact Assessment Results

**Figure 7:**
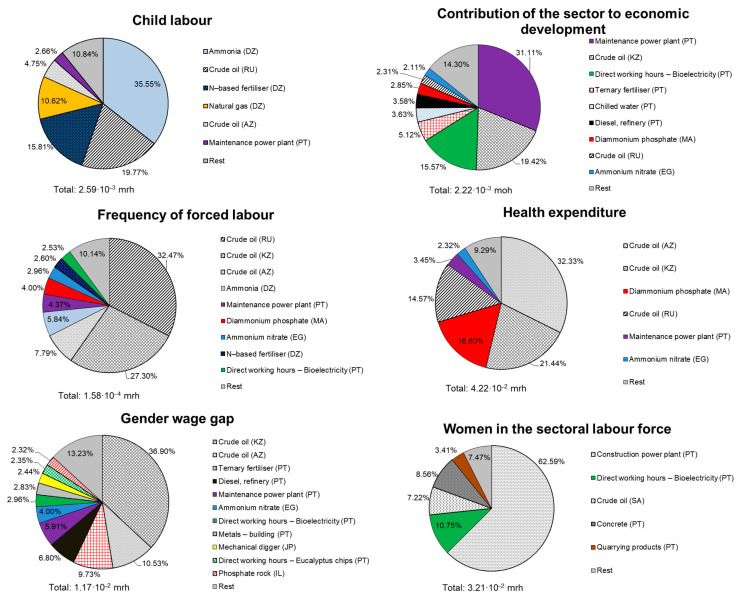
S-LCIA results of the bioelectricity system based on fluidised-bed technology (values per FU).

S-LCIA results were calculated by implementing the S-LCI data in the openLCA software [44] and using the PSILCA framework [42]. Figure 7 shows the quantification of the social life-cycle profile and the relative contribution of each unit process to each social indicator for the biomass-to-electricity system based on fluidised-bed furnace technology. Undesirable social impacts were measured in medium risk hours (mrh), whereas the positive impact indicator (contribution to economic development) was measured in medium opportunity hours (moh). It should be noted that the label “rest” contains all processes with contributions below a threshold value of 2% under each indicator. According to the results in Figure 7, the social hotspots of the bioelectricity system based on fluidised-bed furnace technology would relate to the following supply chain areas: extraction of crude oil, production of fertilisers, and power plant infrastructure (both construction and maintenance). It should be noted that according to the methodological approach followed in this work, this social hotspot identification relies on the cumulative amount of working hours within specific sectors and countries with non-null risk levels [42].

Crude oil extraction was identified as the area with the greatest contribution for three out of six social indicators: gender wage gap, health expenditure, and forced labour. The corresponding contribution percentages under these indicators were estimated at 48% for gender wage gap and above 65% for health expenditure and forced labour. These high contributions to unfavourable social impacts were found to be linked to the mix of countries (and their corresponding sector-specific risk levels) that supply crude oil to Portugal: Russia, Azerbaijan, Saudi Arabia, and Kazakhstan. Concerning the child labour indicator, the production of N-based fertiliser and its main associated compound (ammonia) was found to account for more than 50% of this social risk. Thus, the import of these commodities along with natural gas (around 11% contribution) from Algeria to Portugal would be responsible for most of the child labour impact due to the high risk level attributed to the Algerian chemical and natural gas sectors. Additionally, crude oil supplied by Russia to Portugal would also contribute significantly to the potential child labour impact.

Finally, power plant construction and maintenance arose as relevant contributors to the indicators of women in the sectoral labour force (unfavourable impact) and contribution to economic development (favourable impact), respectively. This is explained by the relatively high number of working hours at the unit process level for these activities. It should be noted that while the indicator “women in the sectoral labour force” refers to a specific gender discrimination risk, the indicator “contribution to economic development” refers to desirable potential effects on job creation, education and training, local investments, and/or research promotion [21,39]. Regarding the latter indicator, bioelectricity production at plant and crude oil extraction would also generate significant added value in their countries of origin (Portugal and Kazakhstan, respectively).

### 3.2. Comparative S-LCA of Biomass-to-Electricity Systems

**Figure 8:**
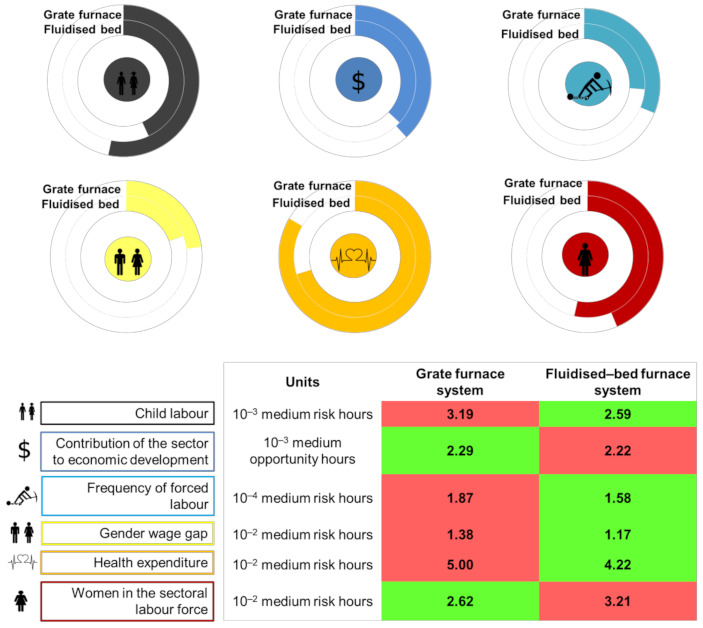
Comparison of the S-LCIA results of both biomass-to-electricity systems (values per FU).

Figure 8 presents the comparison of the S-LCIA results per FU (1 kWh of electricity) for bioelectricity based on fluidised-bed furnace technology (Section 3.1) and bioelectricity based on grate furnace technology [19]. Consistent methodological choices in both systems (regarding e.g., boundaries and cut-off rules) allow for a robust comparative analysis under each separate social life-cycle indicator [21]. A key finding from this comparative analysis is that the implementation of fluidised-bed furnace technology could lead to reductions of 15–19% in all the evaluated negative social impacts, except for the indicator “women in the sectoral labour force” (19% increase), while contribution to economic development (positive impact) would be reduced by only 3%.

The use of the same biofeedstock leads to the identification of similar hotspots under the following social indicators: gender wage gap, health expenditure, child labour, and forced labour. Under the remaining social indicators, the implementation of a more efficient conversion technology increases the leading role of the activities related to the infrastructure of the power plant (both construction and maintenance). An increased efficiency means a reduction in feedstock and labour requirements at the expense of higher capital investments, leading to an increased relevance of the plant’s capital goods as well as, in general, to an enhanced social life-cycle performance of bioelectricity. Coming back to the research question raised in Section 2, this study shows—from an analytical perspective—that technological improvements in biomass-to-electricity systems can lead to a reduction in social risks across the supply chain. Nevertheless, additional studies—not limited to the field of bioelectricity—should be conducted to gain further insight into the finding that the implementation of technological improvements aimed at resource efficiency has a large potential for enhancing the social life-cycle performance of a product system, thus promoting social responsibility through the supply chain. 

### 3.3. Broadening the Discussion to Sustainability

**Figure 9:**
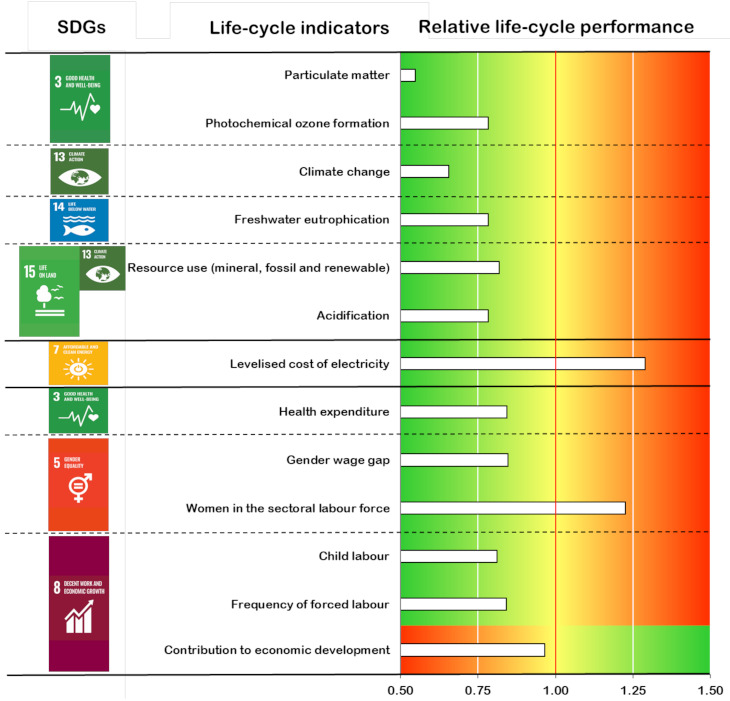
Life-cycle sustainability indicators of bioelectricity based on fluidised-based furnace technology relative to those of bioelectricity based on grate furnace technology.

This section enlarges the comparison between the biomass-to-electricity systems by exploring a joint interpretation and benchmarking of life-cycle sustainability indicators. Even though this enlarged discussion is not intended to be a fully robust life cycle sustainability assessment, there is full consistency within each of the sustainability dimensions as well as between the economic and social dimensions. Furthermore, methodological variations in the environmental dimension with respect to the economic and social ones (e.g., the role of capital goods was not considered in the environmental indicators in the original study [22]) involve a negligible influence on the comparison results. Hence, the joint interpretation presented in this section (Figure 9) allows finding valuable lessons on how technological improvements can affect the life-cycle sustainability performance of energy systems. Environmental results in terms of particulate matter, photochemical ozone formation, climate change, freshwater eutrophication, resource use, and acidification were retrieved from da Costa et al. [22], while the levelised cost of electricity was used as the economic life-cycle indicator according to the approach in Valente et al. [45] and the data in Martín-Gamboa et al. [19] and Section 2.3. Social results correspond to those from Section 3.2. In Figure 9, the horizontal bars falling into the green area indicate a better performance of bioelectricity based on fluidised-bed furnace technology, while horizontal bars reaching the red area indicate a better performance of bioelectricity based on grate furnace technology. The yellow area implies a similar performance of both options.

As shown in Figure 9, bioelectricity from the fluidised-bed furnace system would outperform that from the grate furnace system in ten out of thirteen life-cycle sustainability indicators. This better performance is especially visible in the environmental dimension, and in particular for the climate change and particulate matter indicators due to reduced direct emissions and improved thermal efficiency. On the other hand, bioelectricity from the grate furnace system would present a better performance only in terms of levelised cost, women in the sectoral labour force, and—to a negligible extent—contribution to economic development. Hence, the increase in the levelised cost when fluidised-bed furnace technology is implemented instead of grate furnace one would be offset by general reductions in environmental and social impacts across the supply chain, paving the way towards a favourable sustainability performance.

The indicators used for this comparative sustainability assessment match specific SDGs, as shown in Figure 9. Within the environmental domain, the proposed indicators relate to four SDGs: SDG3 on good health and well-being, SDG13 on climate change, SDG14 on life below water, and SDG15 on life on land. In the economic realm, a connection is established with SDG7 on affordable and clean energy. Concerning the social domain, links with three SDGs were found: SDG3 on good health and well-being, SDG5 on gender equality, and SDG8 on decent work and economic growth. In this sense, the adoption of a more efficient biomass-to-electricity technology would help progress in alignment with SDGs. This type of sustainability reporting presents a high added value for policy-makers and company actors, supporting decision-making processes based on informed and robust choices. Moreover, ongoing initiatives to develop a clear linkage between SDGs knowledge and life-cycle approaches may further contribute to boosting the effectiveness of informed decision-making and strategy development [46].

## 4. Conclusions

A comparative S-LCA of two biomass-to-electricity systems located in Portugal was conducted in this work. Thus, the present study contributes to filling the gap regarding the potential social impacts of bioelectricity, and it sheds light on the potential social effects associated with technological enhancement in energy systems. First, the separate S-LCA of bioelectricity from a fluidised-bed system led to identifying three supply chain areas where social risks would be concentrated: crude oil extraction, fertiliser production, and power plant infrastructure (both construction and maintenance). Crude oil supplied by Russia, Azerbaijan, Saudi Arabia, and Kazakhstan to Portugal would be the major contributor to gender wage gap, health expenditure, and forced labour. The production of N-based fertiliser, ammonia, and natural gas from Algeria would account for the highest contribution to child labour, while power plant construction and maintenance would be a social hotspot in terms of women in the sectoral labour force.

The comparison between bioelectricity from a fluidised-bed furnace system and that from a grate furnace system shows that the implementation of fluidised-bed furnace technology as a more efficient solution could lead to a reduction of 15–19% in all the evaluated negative social impacts (with the exception of women in the sectoral labour force). Concerning the location of impacts within the supply chain of each system, the use of the same biofeedstock led to equivalent hotspots in terms of gender wage gap, health expenditure, child labour, and forced labour. Under the remaining indicators (women in the sectoral labour force and contribution to economic development), the implementation of a more efficient conversion technology increased the leading role played by the construction and maintenance of the power plant. The conclusion on the preference for bioelectricity based on fluidised-bed furnace technology over that based on grate furnace technology was further stressed when broadening the assessment to a life-cycle sustainability perspective, where the former outperformed the latter in ten out of thirteen sustainability indicators. Nevertheless, since the study is limited to two specific case studies on bioenergy, further works are required in order to strengthen the finding on the link between efficiency and sustainability in energy systems. Overall, this study provides a valuable starting point that opens up a research line to ascertain the potential of technological improvements when it comes to effectively mitigating social and environmental impacts, leveraging the ability of life-cycle approaches for informed decision-making and sustainability reporting.

## Figures and Tables

**Table 1:** Main features of the energy conversion stage of the biomass-to-electricity systems.

Feature	Units	Grate Furnace	Fluidised Bed
Feedstock	-	Eucalyptus logging residues	Eucalyptus logging residues
LHV of feedstock (dry basis) ^1^	MJ/t	17.5	17.5
Nominal power	MW_e_	12.5	25
Annual electricity production ^2^	MWh	62,478	85,387
Thermal efficiency ^2^	%	20	25
Typical temperature in the furnace ^3^	°C	900–1100	750–950
Typical gas velocity in the furnace ^3^	m/s	2.4–3.0	1.0–6.0
Typical combustion efficiency ^3^	%	94–97	~99
Eucalyptus chips consumption (dry basis) ^2^	kg/kWh	1.1	0.9
Personnel ^4^	workers	16	16
Annual working hours ^4^	h/worker	1840	1840

^1^ Based on [34]; ^2^ based on [22]; ^3^ based on [35]; ^4^ based on [19].

## Abbreviations

**Abbreviation**	**Description**
FU	Functional unit
IEA	International Energy Agency
IRR	Internal rate of return
LCA	Life cycle assessment
LCC	Life cycle costing
LCI	Life cycle inventory
moh	Medium opportunity hours
mrh	Medium risk hours
NPV	Net present value
PBP	Payback period
S-LCA	Social life cycle assessment
S-LCI	Social life cycle inventory
S-LCIA	Social life cycle impact assessment
SDG	Sustainable development goal
UNEP	United Nations Environment Programme
